# Inpatient mortality rates during an era of increased access to HIV testing and ART: A prospective observational study in Lilongwe, Malawi

**DOI:** 10.1371/journal.pone.0191944

**Published:** 2018-02-07

**Authors:** Mitch M. Matoga, Nora E. Rosenberg, Christopher C. Stanley, Sylvia LaCourse, Charles K. Munthali, Dominic P. Nsona, Bryce Haac, Irving Hoffman, Mina C. Hosseinipour

**Affiliations:** 1 University of North Carolina Project, Lilongwe, Malawi; 2 University of North Carolina, Chapel Hill, North Carolina, United States of America; 3 University of Washington, Seattle, Washington, United States of America; 4 Kamuzu Central Hospital, Lilongwe, Malawi; 5 Lighthouse Trust, Lilongwe, Malawi; 6 University of North Carolina, Chapel Hill, North Carolina, United States of America; University of New South Wales, AUSTRALIA

## Abstract

**Background:**

In the era of increased access to HIV testing and antiretroviral treatment (ART), the impact of HIV and ART status on inpatient mortality in Malawi is unknown.

**Methods:**

We prospectively followed adult inpatients at Kamuzu Central Hospital medical wards in Lilongwe, Malawi, between 2011 and 2012, to evaluate causes of mortality, and the impact of HIV and ART status on mortality. We divided the study population into five categories: HIV-negative, new HIV-positive, ART-naïve patients, new ART-initiators, and ART-experienced. We used multivariate binomial regression models to compare risk of death between categories.

**Results:**

Among 2911 admitted patients the mean age was 38.5 years, and 50% were women. Eighty-one percent (81%) of patients had a known HIV status at the time of discharge or death. Mortality was 19.4% and varied between 13.9% (HIV-negative patients) and 32.9% (HIV-positive patients on ART ≤1 year). In multivariable analyses adjusted for age, sex and leading causes of mortality, being new HIV-positive (RR = 1.64 95% CI: 1.16–2.32), ART-naive (RR = 2.28 95% CI: 1.66–2.32) or being a new ART-initiator (RR = 2.41 95% CI: 1.85–3.14) were associated with elevated risk of mortality compared to HIV-negative patients. ART-experienced patients had comparable mortality (RR = 1.33 95% CI: 0.94–1.88) to HIV-negative patients.

**Conclusion:**

HIV related mortality remains high among medical inpatients, especially among HIV-positive patients who recently initiated ART or have not started ART yet.

## Introduction

Marked and sustained reductions in opportunistic disease and AIDS-related death have been observed world-wide as a consequence of increased access to HIV testing and extensive use of antiretroviral therapy (ART) [1–4]^.^ These health benefits have been observed across diverse patient populations, leading to reduced hospitalization and mortality from HIV/AIDS related illnesses [5–9] and increased life expectancy [10].

The Malawi Ministry of Health (MOH) launched a major initiative to increase access to ART using a public health model in 2004 and introduced provider initiated testing and counseling (PITC) for all patients accessing healthcare in 2011 [11]. By 2011, 67% of eligible adults and children were on ART [12]. In 2011, Malawi guidelines called for ART initiation at CD4 count < = 350 cells/ μL or WHO stage 3 or 4 conditions and for those ineligible, quarterly assessments for co-trimoxazole and CD4 count. Kamuzu Central Hospital (KCH), a tertiary care center for central Malawi, adopted PITC in its medical and surgical wards in November 2011.This program resulted in an almost 2-fold increase in HIV case-finding by the time of discharge [13]. Increased case-finding allows patients to access HIV care which includes early diagnosis and treatment of opportunistic infections and early ART initiation. These treatments are known to reduce mortality from AIDS-related illnesses and transmission to uninfected partners [14].

Despite a recent study conducted at a tertiary care center in southern Malawi within an era of PITC and ART scale-up [15], detailed descriptions of the spectrum of disease and mortality among hospitalized HIV-positive and HIV-negative patients are generally lacking after the introduction of PITC and ART roll-out in Malawi. Furthermore, descriptions of mortality among hospitalized HIV-positive patients by previous HIV diagnosis knowledge, ART status and ART duration are lacking after the early expansion of the nation’s ART program through ART initiation at higher CD4 count. We sought to evaluate trends in morbidity and mortality among patients hospitalized in the medical wards of a large referral hospital in a low resource setting with a maturing ART program and post PITC introduction. We described the causes of morbidity and mortality, determined mortality rates, and relationship between HIV and ART status and mortality among adults admitted to the medical wards at Kamuzu Central Hospital. We categorized patients as HIV-negative, new HIV-positive, ART-naïve, new ART-initiators and ART-experienced.

## Materials and methods

We conducted a prospective observational study of all medical admissions at KCH in Lilongwe, Malawi, between November 2011 and April 2012. KCH serves as the tertiary care center for the central region of Malawi to a population of approximately seven million people. At the time of the study, the hospital’s medical wards had approximately 117 beds and approximately 46,000 patients were evaluated annually and assessed for admission. Patients were referred from primary care units within Lilongwe, secondary care units from surrounding districts and self-referrals. All patients were reviewed at the outpatient department (OPD) which was staffed by Medical Assistants and Clinical Officers for a quick assessment of the patients’ clinical condition and commencement of emergency care where necessary. From the OPD, patients whose clinical condition needed more attention were sent to the medical “short-stay” (observation) ward where further management was offered by the on-call team which usually consisted of about two interns, one clinical officer, two registrars and one consultant. Patients whose conditions warranted admission were sent to the medical wards, which included a four-bed high-dependency unit (HDU) while the rest of the patients were discharged from the “short-stay” ward after receiving appropriate care. Patients were treated separately in male and female wards.

Our study population consisted of all individuals (aged ≥ 15 years) who were admitted to the adult medical wards, including the HDU. Patients under 15 years of age were admitted to the pediatric wards. There were no exclusion criteria. At the time of admission, patients were offered HIV testing in the medical pre-admission ward. If patients did not get tested pre-admission, they were offered HIV testing in the medical wards. HIV counselors approached all patients and evaluated them for evidence of previous HIV testing. Patients with no documented HIV test results or documented negative test from more than three months prior were offered and received PITC unless they explicitly refused. Two HIV counselors conducted pre-and post-test counseling services and recorded results in patient files and in MOH HIV testing logs. Counselors were available Monday through Friday from 8am to 4pm and one counselor worked Saturday mornings. Patients were admitted until they were discharged, died or absconded.

We collected clinical course and outcome data using a standard admission form. The form included patient characteristics such as age, gender, HIV and ART status, physical exam findings, admission diagnoses, final discharge diagnoses, and patient outcomes of death, discharge or absconding. The admitting clinician (physician or clinical officer) recorded information on the form while the discharging clinician recorded the final diagnoses. Once a patient had a final outcome, a ward clerk transferred information from the admission form onto a data collection form and which was entered into a Microsoft Office Access version 14.0 database by a data assistant. Any information that was missing from the admission form was entered as ‘unknown’ in the database. To capture all inpatient admissions, a research assistant compared data in the spread sheet with available patient forms and the number of patients recorded in the admission logs weekly. In the event that an admission was not entered into the spreadsheet, the data were recorded at the time of discovery. During the study period, KCH had a laboratory with; hematology, microbiology, parasitology, biochemistry, serology and blood bank departments. However, due to limited availability of resources, different tests were not readily available. This resulted in clinicians making presumptive diagnosis which was evidenced by multiple final diagnoses per patient and presumptive treatment. The standard treatment for common conditions was: intravenous (IV) quinine for severe malaria; IV benzyl-penicillin and gentamycin or ceftriaxone for pneumonia; IV chloramphenicol and gentamycin or ceftriaxone for sepsis; high dose ceftriaxone for bacterial meningitis; high dose fluconazole for cryptococcal meningitis; and rifampicin, isoniazid, pyrazinamide and ethambutol for tuberculosis (TB).

In order to assess the impact of PITC and ART roll-out, we categorized patients as HIV-negative, HIV-positive, and unknown HIV status by the time of final outcome. HIV-positive patients were classified as “new HIV-positive” if they tested positive during admission and “known positive” if they had a previously documented HIV-positive test. Known positives were sub-divided by ART status–ART-naïve (not on ART), new ART-initiators (ART ≤ 1 year) and ART-experienced (ART > 1 year).

For the purposes of our analysis, we used the final discharge diagnoses for all patients unless they were missing in which case the admission diagnoses were used. We included HIV-negative, new HIV-positive, ART-naïve, new ART-initiators and ART-experienced patients in our analyses and identified the most common causes of morbidity and mortality, and compared mortality across these categories. We excluded patients with unknown HIV status or unknown ART status or unknown ART duration. Categorical variables were compared using Pearson’s chi-squared tests. Risk ratios (RR) and 95% confidence intervals comparing mortality by HIV and ART status for the most common diagnoses were calculated using generalized linear models with a binomial family and log function. We controlled for age, sex, and leading causes of mortality (pneumonia, sepsis, anemia, malaria, cryptococcal meningitis, and acute and chronic gastroenteritis) in adjusted models. All data analyses were conducted using Stata SE version 12.0 (Stata Corp., College Station, TX). Statistical significance was considered at a two-sided α-level of 0.05.

Both the National Health Science Research Committee of Malawi and the University of North Carolina Internal Ethics Review Board approved this study.

## Results

### Baseline characteristics

**Fig 1:**
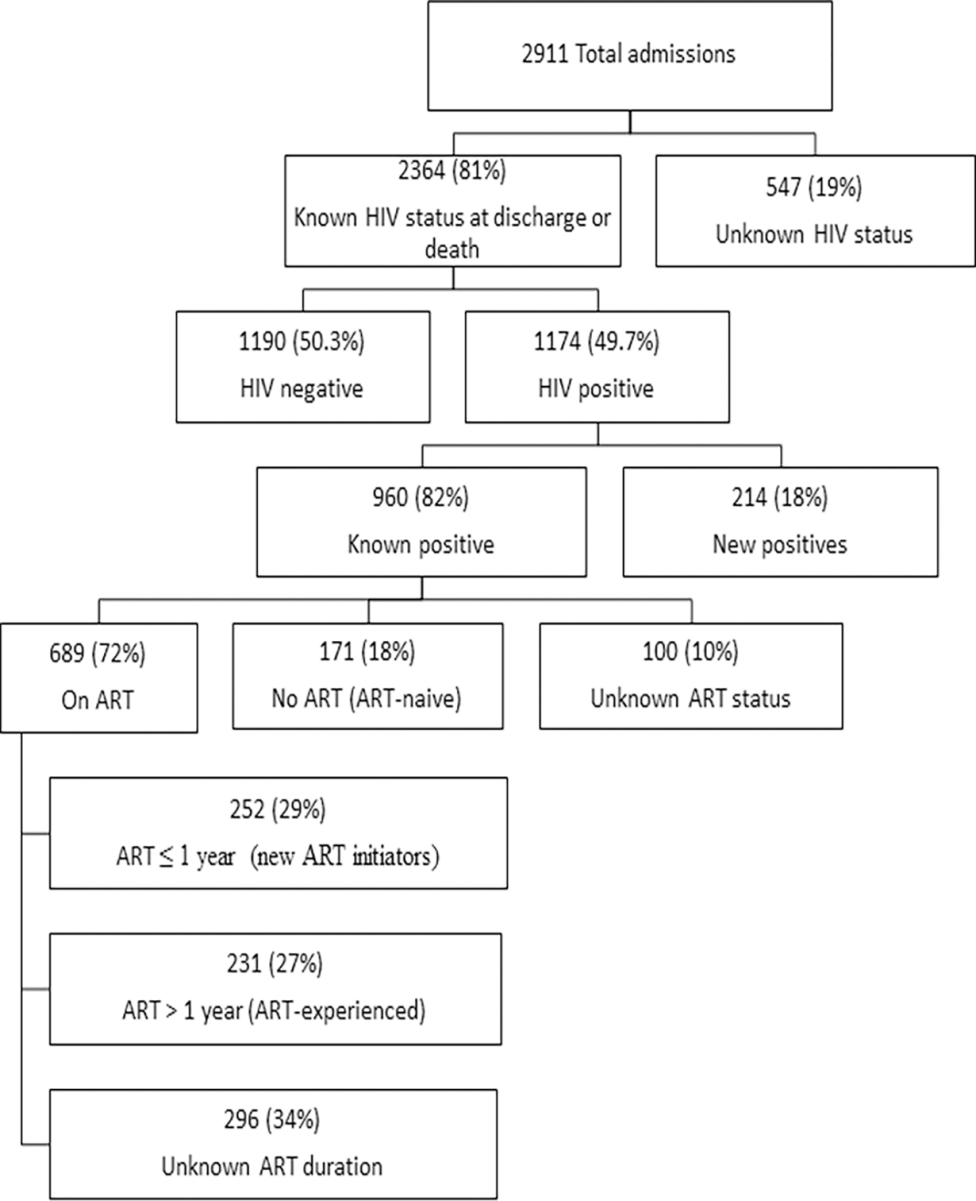
Distribution of inpatient HIV and ART status on the medical wards at KCH.

Among 2911 patients admitted to the KCH medical ward over 6 months, 1454 (49.9%) were men and 1457 (50.1%) were women (Table 1). The mean age was 38.5 years (SD ±16.5). The median time to discharge for survivors was 3 days (IQR: 1, 5) while the median time to death was 2 days (IQR: 1, 5). Most (81%) had a known HIV status by the time of discharge or death and the rest (19%) did not. The prevalence of HIV was 40.3% (1174) among admitted patients. Among those with HIV infection, 960 (82%) were known positives and 214 (18%) were new HIV-positives. Of the 960 known positives prior to admission, 860 (73%) patients had documentation of ART status of which 689 (72%) patients were already on ART, 171 (18%) were ART-naive and 100 (10%) had an unknown ART status. Of those on ART, 252 (29%) patients were new ART-initiators (≤ 1 year), 231 (27%) were ART-experienced (> 1 year) and 206 (24%) had an unknown duration on ART (Fig 1). Among the five HIV status categories of our analysis, 1190 (57.8%) were HIV-negative, 214(10.4%) were new HIV-positives, 171(8.3%) were ART-naive, 252(12.2%) were new ART-initiators and 231(11.3%) were ART-experienced.

**Table 1:** Baseline characteristics by HIV and ART status among medical inpatients.

Characteristic	HIV-negative N = 1190	New HIV-positive N = 214	ART-naïve N = 171	New ART-initiators N = 252	ART-experienced N = 231	P-value Pearson chi-square
**Sex**						0.011
Male	50.0%	52.3%	52.1%	48.8%	38.1%	
Female	50.0%	47.7%	47.9%	51.2%	61.9%	
**Age**						<0.001
≤19	14.8%	3.4%	2.4%	1.6%	4.8%	
20–29	24.7%	32.5%	25.9%	24.1%	14.0%	
30–39	17.3%	33.5%	47.6%	40.6%	45.0%	
40–49	9.3%	18.0%	17.5%	20.5%	21.0%	
≥50	33.9%	12.6%	6.6%	13.2%	15.2%	
**Number of Diagnoses**						0.019
1	64.7%	59.4%	54.4%	60.0%	58.0%	
2	27.1%	29.4%	32.2%	28.6	29%	
≥3	5.7%	10.3%	11.1%	7.1%	8.7%	
**Duration of Hospital stay**						0.509
0–3	57.4%	53.7%	50.3%	61.1%	56.7%	
4–7	23.7%	23.2%	26.8%	26.2%	26.6%	
>7	18.9%	23.1%	22.9%	12.7%	16.7%	

### Causes of mortality and morbidity

The overall mortality among admitted patients was 19.4% (565/2911). Of all deaths, the five leading causes of mortality were pneumonia (15.9%), bacterial meningitis (14.9%), malaria (12.9%), anemia (12.9%) and TB (11.3%). Mortality among HIV-positive patients was 23.7% and it was specifically high among new ART-initiators (32.9%) and ART-naïve patients (26.3%). Bacterial meningitis (52.8%) and TB (39.9%) and cryptococcal meningitis (38.5%) had the highest case fatality rates (Table 2). Infectious diseases were the leading cause of morbidity. Among all admitted patients, the most common causes of morbidity were malaria (22.9%), pneumonia (12.2%), anemia (10.6%), sepsis (10.4%) and acute gastroenteritis (6.4%). Across the five patient categories, malaria and pneumonia were the most common causes of morbidity (Table 2). WHO stage 3 and 4 conditions accounted for 56.0% of admissions among HIV positive patients.

**Table 2:** Mortality by HIV and ART status among medical inpatients.

	HIV-Negative (N = 1190)	New HIV-Positive (N = 214)	ART-Naïve (N = 171)	New ART-Initiators (N = 252)	ART-Experienced (N = 231)
**Total Percent Survived**	**94.6**	**81.8**	**73.7**	**67.1**	**84.4**
**Total Percent Died**	**5.4**	**18.2**	**26.3**	**32.9**	**15.6**
**Disease Condition**					
Malaria (N, %)	296, 24.9	69, 32.2	38, 32.2	25, 9.9	33, 14.3
% Survived	93.9	85.5	86.8	80.0	90.9
% Died	6.1	14.5	13.2	20.0	9.1
Pneumonia (N, %)	100, 8.4	33, 15.4	37, 21.6	48, 19.0	32, 13.9
% Survived	77.0	81.8	70.3	79.2	84.4
% Died	33.0	18.2	29.7	20.8	15.6
Anemia (N, %)	122, 10.3	24, 11.2	29, 17.0	26, 10.3	29, 12.6
% Survived	86.1	75.0	65.5	69.2	86.2
% Died	13.9	25.0	34.5	30.8	13.8
Sepsis (N, %)	119, 10.0	18, 8.4	30, 17.5	24, 9.5	27, 11.7
% Survived	85.7	88.9	70.0	70.8	81.5
% Died	14.3	11.1	30.0	29.2	18.5
Acute Gastroenteritis (N, %)	60, 5.0	26, 12.1	13, 7.6	14, 5.6	15, 6.5
% Survived	90.0	84.6	92.3	85.7	100.0
% Died	10.0	15.4	7.7	14.3	0.0
Congestive Heart Failure (N, %)	112, 9.4	5, 2.3	4, 2.3	8, 3.2	9, 3.9
% Survived	79.5	60.0	50.0	37.5	66.7
% Died	20.5	40.0	50.0	62.5	33.3
Bacterial Meningitis (N, %)	38, 3.2	25, 11.7	12, 7.0	23, 9.1	16, 6.9
% Survived	63.2	56.0	16.7	39.1	68.7
% Died	35.8	44.0	83.3	60.9	31.3
Pulmonary TB (N, %)	28, 2.4	9, 4.2	17, 9.9	32, 12.7	10, 4.3
% Survived	53.6	77.8	70.6	50.0	50.0
% Died	46.4	22.2	29.4	50.0	50.0
Extra-Pulmonary TB (N, %)	15, 1.3	2, 0.9	3, 1.8	8, 3.2	6, 2.6
% Survived	86.7	50.0	33.3	50.0	100.0
% Died	13.3	50.0	66.7	50.0	0.0
Kaposi’s Sarcoma (N, %)	3, 0.3	3, 1.4	2, 1.2	11, 4.4	12, 5.2
% Survived	100.0	100.0	50.0	81.8	75.0
% Died	0.0	0.0	50.0	18.2	25.0
Cryptococcal Meningitis (N, %)	0, 0.0	6, 2.8	4, 2.3	8, 3.2	3, 1.3
% Survived	N/A	50.0	75.0	62.5	66.7
% Died	N/A	50.0	25.0	37.5	33.3
Other (N, %)	427, 35.9	43, 20.1	34, 19.9	70, 27.8	77, 33.3
% Survived	85.7	79.1	76.5	68.6	85.7
% Died	14.3	20.9	23.5	31.4	14.3

Note: Percentage of diagnoses add up to >100% because some patient had multiple diagnoses

N/A: Not applicable

### HIV and mortality

In unadjusted analyses, being known positive and ART-naïve (RR = 1.89 95% CI: 1.41–2.52) or being known positive and a new ART-initiator (RR = 2.19 95% CI: 1.73–2.77) were associated with higher risk of mortality compared to being HIV-negative (Table 3). Being a new HIV-positive (RR = 1.31 95% CI: 0.95–1.79) exhibited a trend towards increased risk of mortality compared to being HIV-negative. ART-experienced patients had a similar risk of mortality (RR = 1.12 95% CI 0.80–1.56) to HIV-negative patients (Table 3). These trends persisted in multivariable analyses adjusted for age, sex and leading causes of mortality (pneumonia, sepsis, anemia, malaria, cryptococcal meningitis, and acute and chronic gastroenteritis). For nearly all of the leading HIV-associated diagnoses (pneumonia, sepsis, meningitis, extra pulmonary TB and Kaposi’s sarcoma), the proportion who died was higher among the ART-naïve patients and new ART-initiators, compared to ART-experienced patients (Table 2). This was true for leading non-HIV associated diagnoses (malaria, anemia, acute gastroenteritis and congestive heart failure).

**Table 3:** Risk of mortality by HIV and ART status among medical inpatients.

Variable	Unadjusted Risk Ratio	[95% CI]	Variable	Adjusted Risk Ratio	[95% CI]
HIV-negative	1	(ref)	HIV-negative	1	(ref)
New HIV-positive	1.31	0.95–1.79	New HIV-positive	1.64	1.16–2.32
ART-naïve	1.89	1.41–2.52	ART-naïve	2.28	1.66–3.12
New ART-initiator	2.19	1.73–2.77	New ART-initiator	2.41	1.85–3.14
ART-experienced	1.12	0.80–1.56	ART-experienced	1.33	0.94–1.88
Sex	0.64	0.55–0.75	Sex	0.66	0.53–0.81
Age	1.01	1.01–1.02	Age	1.01	1.01–1.02
Pneumonia	1.38	1.14–1.68	Pneumonia	0.91	0.69–1.21
Sepsis	1.08	0.85–1.36	Sepsis	1.07	0.78–1.47
Malaria	0.51	0.40–0.64	Malaria	0.50	0.36–0.71
Anemia	1.27	1.03–1.58	Anemia	1.00	0.73–1.37
Cryptococcal Meningitis	2.00	1.22–3.27	Cryptococcal Meningitis	1.48	0.86–2.56
Kaposi’s sarcoma	1.07	0.62–1.87	Kaposi’s sarcoma	0.77	0.38–1.56
Acute Gastroenteritis	0.45	0.29–0.72	Acute Gastroenteritis	0.43	0.23–0.80
Chronic Gastroenteritis	0.90	0.37–2.19	Chronic Gastroenteritis	1.02	0.39–2.70

## Discussion

In our review of mortality in a large tertiary facility in Malawi, mortality was extremely high, but varied by HIV status, HIV diagnosis awareness prior to admission, ART status, and ART duration. Patients who knew they were HIV-positive and ART-naïve, and new ART-initiators, were more than twice as likely to die compared to HIV-negative patients. New HIV-positive patients also had a trend towards increased risk of mortality. ART-experienced patients had a similar risk of mortality compared to HIV-negative patients.

This is one of the first studies to analyze causes of admissions and mortality in Malawi in an era of PITC implementation and early ART scale-up. Compared to a similar study in this facility conducted in 2008–9 prior to PITC, [3], a higher proportion of patients knew their HIV status by discharge (81.1% versus 37.3%). Compared to a similar study conducted in Blantyre Malawi within era of ART scale-up and PTIC (2013–14), 56.4% of medical inpatients knew their HIV status by discharge [15]. Our results signify that in only three years, inpatient HIV testing had markedly improved. We demonstrated that PITC, if properly implemented, can improve the rates of inpatient HIV testing just as reported in other countries within the region [16]. The HIV prevalence of 40.3% in our study was comparable to that found by La Course et al during role out of PITC (39.3%) at the same hospital [13]. Similarly, the HIV prevalence rate was also comparable to a study done in Blantyre, Malawi within an era of PITC and ART scale-up where they found 4551/10191 (44.7%) positive patients [15]. However, the prevalence rate was lower compared to some countries within the region; South Africa (60.1%) [17] and Botswana (47.5%) [18]. The HIV prevalence rates among inpatients in Malawi has improved compared to the pre-ART era (70%) [19] highlighting the progress in the country’s ART program.

The overall mortality rate was somewhat higher (19.4% versus 14.6%), but mortality among HIV-infected patients was similar (23.7% versus 24.2%) compared to prior to PITC [3]. Compared to studies within the country and region, our medical ward mortality rate was similar to that reported in South Africa (17%) [17], Botswana (23%) [18] and Blantyre, Malawi (22.7%) [15] within a period of PITC and early ART scale-up. The mortality rate among HIV positive patients in our study was lower than that found in a recent systematic review and meta-analysis of 31% within Africa [20] but was somewhat consistent with a mortality rate range of 24.2% to 44% within SSA reported in another study [16]. The sustained high mortality rate among HIV-infected patients may reflect incomplete maturation of Malawi’s ART program, combined with HIV-infected patients presenting to the hospital at a late stage of disease. Late presentation to the hospital has been reported to be associated with higher mortality [16].

Known positive patients who were ART-naïve had high mortality rates. This patient population was predominantly burdened by opportunistic infections (OIs) specifically TB, pneumonia, sepsis, and meningitis. OIs were associated with a higher risk of mortality in Blantyre Malawi [15]. The increased risk of mortality, along with a high prevalence of opportunistic infections, suggests severe immune suppression at the time of presentation. Our results suggest that these ART-naïve patients may have been tested but not in care (poor linkage) or in care with inadequate monitoring practices to promptly identify disease progression or they may have dropped out of care (poor retention) until developing an opportunistic infection requiring hospital admission. In Tanzania, it was found that many patients were lost to follow-up after hospitalization and this was associated with higher mortality as patients re-presented to the hospitals later with advanced HIV disease [16]. Effort needs to be put into early linkage to care and retention in care in order to achieve the UNAIDS 90-90-90 targets [21]. The recent WHO recommendation for universal access to ART regardless of CD4 count may be one way of addressing this set of challenges [22].

New ART-initiators also had elevated mortality rates. Similar to ART-naïve patients, opportunistic infections were the biggest burden in this patient population. Early mortality within the first year of treatment is a challenge in most ART programs in resource-limited settings with mortality ranging between 7% and 17% [23,24]. The majority of these deaths occur within the first three months of treatment [25,26] and are attributed to opportunistic infections [2,27]. Early mortality is associated with several factors including low baseline CD4 count, anemia, low BMI, and advanced WHO clinical stage which are known to be strong predictors of mortality [23]. Early mortality may also be related to Immune Reconstitution Inflammatory Syndrome (IRIS) which occurs among new ART-initiators, particularly among those with low CD4 counts [26]. IRIS may include acute worsening of known infections or unmasking of previously unrecognized, life-threatening opportunistic infections within the first six months of treatment [26]. These results suggest the need for early linkage to care and early ART initiation to prevent severe immunosuppression with the risk of opportunistic infections and IRIS. Late ART initiation remains a big concern world-wide and within the regions as many studies have reported very low CD4 counts and advanced WHO stage at the start of ART treatment [20,28]. Early linkage to care and early ART initiation may be one of the ways to ensure that more people who are tested positive for HIV start treatment thereby contributing towards achieving the second ninety of the UNAIDS targets. Further, universal access to ART as recommended by WHO will be one of the ways to address this public health problem.

ART-experienced patients did not have higher mortality rates compared to HIV-negative individuals. However, among those who did die, HIV-related conditions, including pneumonia, sepsis, tuberculosis (TB) and meningitis were the most common causes of mortality. HIV-related conditions are the commonest cause of mortality among HIV-infected individuals world-wide [20]. There have been reports of mortality benefits of ART in the country and worldwide [8,9,29]. Similarly, our results reflect a success in the ART program as more people initiated and maintained on treatment have better survival rates. However, the comparable mortality between ART-experienced patients and HIV-negative patients may have been affected by the lack of data on ART history. Nonetheless, these results underscore the continued need for treatment adherence and retention in care, the third ninety of the UNAIDS targets, to prevent disease burden from OIs while on ART. Developing an OI while on ART > 1 year may suggest treatment failure, potentially due to poor adherence or HIV drug resistance.

In our study, the risk of death did not vary by age and diagnoses; pneumonia, sepsis, malaria, anemia, cryptococcal meningitis, kaposis sarcoma and gastroenteritis. Compared to a study done in Blantyre, age >55 years and diagnoses (candidiasis, gastroenteritis, kidney disease, meningitis and cancers) were associated with higher risk of death [15]. Our findings could be explained by the relatively small sample size compared to the Blantyre study which looked at over 10,000 patient records. Most diagnoses were made on clinical grounds with limited radiographic and laboratory diagnostics. For instance, malaria was one of the most common causes of morbidity and mortality in our study. Due to limited microbiology testing in our setting, the term “malaria” often encompass many febrile illnesses. Compared to the Blantyre study done in a larger tertiary hospital with robust microbiological support, malaria was not among the most common causes of morbidity and mortality [15]. Despite the fact that our study was conducted during a high malaria transmission season, the large burden of malaria shown in our study highlights the lack of microbiological diagnostics.

We used discharge diagnoses, as opposed to initial diagnoses, in order to capture work-up done during hospitalization and to better classify true diagnoses. However, in some cases work-up was incomplete at the time of death or discharge thereby resulting in inaccuracies in classification of diagnoses and causes of mortality. High patient volumes at KCH may have also led to clinicians making expedited diagnoses that may have been inaccurate. Due to a scarcity of medical specialists in our settings, in cases where senior clinicians were not available, junior clinicians who may have lacked clinical experience may have contributed to the inaccuracy of clinical diagnoses. The lack of diagnostics may have also contributed to mortality in cases where the actual diagnosis was not known and patients were treated presumptively or inappropriately. However, all of these factors would have applied equally to each of the patient populations evaluated such that the study results related to HIV status, mortality and diagnosis should not be affected.

Absence of immunologic, virologic, and clinical staging of HIV limited our ability to accurately characterize the HIV disease state at the time of presentation for each of the patient groups. Additionally, we were not able to study the role of CD4 count and viral load which are known risk factors for mortality [2,4,26,30].

Persons with unknown HIV status, ART status, or ART duration were excluded from the analyses. Approximately one-third of patients on ART did not have ART data on their admission forms and a large proportion of the remaining HIV-positive patients did not have complete ART history which resulted in few ART data available for analysis. As the country’s HIV program continues to grow, future research will be warranted to understand the morbidity and mortality trends among hospitalized patients. Furthermore, research detailing better diagnostics, triage procedures and patient characteristics associated with mortality may help increase the accuracy of diagnoses and identify at-risk populations.

Our results emphasize the importance of the UNAIDS 90-90-90 targets [21]. They underscore the need for increasing HIV testing to identify HIV infection earlier, improving linkage to and retention in care, and enhancing patient ART adherence. In summary, we demonstrated that mortality due to HIV-related conditions remained common among medical inpatients. Many patients were diagnosed with HIV late and initiated treatment late. Implementation research to find better ways to increase HIV testing, improve linkage to care and retain people in care while adhering to treatment are required.
